# Helminth lifespan interacts with non-compliance in reducing the effectiveness of anthelmintic treatment

**DOI:** 10.1186/s13071-018-2670-6

**Published:** 2018-01-31

**Authors:** Sam H. Farrell, Roy M. Anderson

**Affiliations:** London Centre for Neglected Tropical Disease Research, Department of Infectious Disease Epidemiology, St Mary’s Campus, Imperial College London, London, W2 1PG UK

**Keywords:** Soil-transmitted helminths, Schistosomiasis, Mass drug administration, Compliance, Systematic non-compliance, Mathematical modelling, Elimination, Transmission interruption

## Abstract

**Background:**

The success of mass drug administration programmes targeting the soil-transmitted helminths and schistosome parasites is in part dependent on compliance to treatment at sequential rounds of mass drug administration (MDA). The impact of MDA is vulnerable to systematic non-compliance, defined as a portion of the eligible population remaining untreated over successive treatment rounds. The impact of systematic non-compliance on helminth transmission dynamics - and thereby on the number of treatment rounds required to interrupt transmission - is dependent on the parasitic helminth being targeted by MDA.

**Results:**

Here, we investigate the impact of adult parasite lifespan in the human host and other factors that determine the magnitude of the basic reproductive number *R*_*0*_, on the number of additional treatment rounds required in a target population, using mathematical models of *Ascaris lumbricoides* and *Schistosoma mansoni* transmission incorporating systematic non-compliance. Our analysis indicates a strong interaction between helminth lifespan and the impact of systematic non-compliance on parasite elimination, and confirms differences in its impact between *Ascaris* and the schistosome parasites in a streamlined model structure.

**Conclusions:**

Our analysis suggests that achieving reductions in the level of systematic non-compliance may be of particular benefit in mass drug administration programmes treating the longer-lived helminth parasites, and highlights the need for improved data collection in understanding the impact of compliance.

## Background

The neglected tropical diseases (NTDs) have become an increasing focus of research over the past decade [1]. The soil-transmitted helminths (STH), a group of nematode parasites, and schistosomiasis, caused by the *Schistosoma* blood flukes, both impose a considerable health burden on poor populations in the developing world [2]. STH infections are acquired through ingestion of eggs or larvae, via unwashed hands, contaminated food or eating utensils, or through penetration of the skin by hookworm larvae. Mild infections may be symptomless but heavier parasite loads are thought to contribute to nutritional deficiencies (which may become severe) as well as serious gastro-intestinal issues, and may cause complications requiring surgical intervention. Schistosomiasis is acquired through contact with contaminated water sources, where infective cercarial larvae penetrate the skin. Infection may induce various symptoms depending on the *Schistosoma* species including anaemia and nutritional deficiencies, intestinal problems and liver damage leading to portal hypertension and a range of symptoms in genital and reproductive systems in both women and men. Low-cost and safe drug interventions are available to treat STH and schistosome infections which enables broad-scale mass drug administration (MDA) programmes to effectively reduce the population burden of infection and concomitant infection. Current WHO treatment guidelines for STH and schistosome infections aim primarily for effective control of morbidity in school-aged children (STH and schistosomes) and women of reproductive age (STH) [2, 3]. Studies to determine the feasibility of interrupting STH transmission by MDA alone in endemic areas are underway [4, 5]. Regardless of the goal of mass treatment, sufficient coverage of the at-risk population is critical to success; WHO targets are to achieve coverage of 75% or more.

Much thought is now being given to the potential role of treatment patterns amongst individuals, as well as population MDA coverage. The focus has begun to shift to the question of individual compliance to treatment over successive MDA rounds, as well as how many are treated. If the same eligible individuals are left untreated over successive rounds, a reservoir of infection may be created hampering the effectiveness of MDA in the population as a whole [6, 7].

We have previously demonstrated that in a stochastic individual based model of helminth transmission and control by MDA, the degree of impact of this systematic non-compliance with treatment on the dynamics of transmission varies between *Ascaris lumbricoides* (roundworm, an STH) and *Schistosoma mansoni* species [8]. We hypothesised that a critical factor in the impact of systematic non-compliance is parasite lifespan, as intuitively a longer adult parasite lifespan in the human host implies a greater impact of untreated individuals. However, a comparative study between the two distinct diseases is not well suited to isolation of the impact of this single factor. In addition, the difficulties of pinpointing time of acquisition and death of individual worms, and the necessity of treating known infection wherever possible, make parasite lifespan in the human host difficult to measure in practice [9]. Careful consideration of its impact is therefore warranted.

In this paper we address these issues directly and investigate dynamic interactions between treatment compliance and estimated parasite lifespan, varying the interdependent variables controlling transmission intensity (the basic reproductive number *R*_*0*_) to gain a clearer picture of the factors affecting the impact of systematic non-compliance. In order to ensure cross-disease applicability of any findings we again investigate transmission of both *A. lumbricoides* and *S. mansoni* parasites.

## Methods

The stochastic individual-based model implementation used in the work motivating this study permits probabilistic forecasts of parasite transmission elimination, as well as allowing for heterogeneity in individual human host compliance with treatment at each round of MDA [8]. This comparative analysis showed different responses to systematic non-compliance between diseases. Here, using a more straightforward deterministic implementation [10, 11], we focus more closely on the critical parameters that interact with compliance in two helminth diseases separately; analysing differences in model outcomes if parasite lifespan were to be longer or shorter than our estimates. The mean prediction of the stochastic model converges to the deterministic model predictions as population sample size increases [8].

Stated in terms of the mathematics of dynamical systems, the models’ long-term behaviour in the absence of disturbance (such as through treatment) is determined by the existence of two attractors. One is the endemic state. The other is a state of zero infection. The dioecious nature of the parasites means that for reproduction a male and female must both be present in a human host, producing a breakpoint in transmission dynamics. Once parasite abundance in the community is reduced below a certain level transmission intensity will be inadequate to sustain the parasite population and will decay to the extinction state without further intervention. The position of the breakpoint is heavily dependent on the degree of parasite aggregation within the human host population, as measured inversely by the negative binomial parameter *k* [12]. As a metric to assess how parasite lifespan in the human hosts impacts the importance of compliance in MDA rounds we choose the minimum number of treatment rounds required to break parasite transmission, i.e. to cross this transmission breakpoint.

A key parameter determining observed epidemiological patterns for all helminth parasites is the basic reproductive number *R*_*0*_. For macroparasites that are dioecious it is defined as the average number of female offspring produced by an adult female worm in the human host that themselves survive to reproductive maturity [12]. By definition, transmission cannot continue if *R*_*0*_ falls below unity in value. However, the magnitude of *R*_*0*_ must be a little above unity in value given the impact of the sexual mating function for dioecious parasites (which are either polygamous or monogamous) on the breakpoint in transmission, the details of which are described in previous publications [10–12].

The magnitude of *R*_*0*_ is determined by many population parameters in the parasite’s life cycle. As shown previously [11], one definition for STH parasites is given in Eq. 1 below:

1 $$ {R}_0=\frac{z\uplambda \uppsi}{\upmu_2\overline{a}}\underset{a=0}{\overset{\infty }{\int }}\rho (a)S(a)\underset{x=0}{\overset{a}{\int }}\beta (x){e}^{-\sigma \left(a-x\right)} dxda $$

The key parameters contributing to *R*_*0*_ include σ which defines the adult parasite death rate in the human host (*1/*σ defines adult parasite life expectancy), the per adult parasite egg production rate in the human host λ, the severity of density dependence in parasite fecundity in the human hosts *z*, the instantaneous rate ψ at which infectious stages (eggs or larvae) enter the environmental reservoir, the mean human host age $\overline{a}$, and the death rate of infectious material in the environmental reservoir *µ*_2_ (where *1/µ*_2_ is infectious stage life expectancy) [10, 11]. The term *S(a)* represents the probability that a human host survives to age *a* (*S(a)* is the survival function), *ρ(a)* defines relative contribution of host age group *a* to the infectious pool of parasite eggs or larvae and ***β****(a)* is the host age-dependent infection rate from this pool of infectious material [11]. The age structure of the host population requires us to consider how human hosts of different ages contribute to and have contact with infectious eggs or larvae in the environment. The age-dependent functions can be estimated from epidemiological data recording age specific parasite intensity and prevalence patterns [11]. Note that while the *R*_*0*_ equation is critical in transmission intensity, these same parameters are found elsewhere in our model and so model behaviour cannot be derived from Eq. 1 alone.

Two compliance settings are examined; *random* compliance (people are treated at random at each round of MDA) and *systematic* non-compliance (people are either always treated at every round, or never). In order to minimise any interactions of compliance-related dynamics with host age, we treat all age groups as having the same coverage (though in reality typically not all age groups are eligible). Treatment is assumed to be annual. Treatment coverage is fixed at 75% of the total population at each round, in accordance with WHO targets [2, 3]. This means that in the systematic non-compliance settings, 25% of the population never receives treatment. The *Ascaris* parasite death rate σ is set at *1/year* (i.e. mean lifespan 1 year) [12] and the *S. mansoni* death rate σ is set at *0.1754/year* (i.e. mean lifespan 5.7 years) [13]. Parameters are otherwise as described by Farrell et al. [8].

Note from the definition of *R*_*0*_ in Eq. 1 that model parameters are interdependent in determining its overall value as a measure of transmission success or intensity. As such, any changes to parasite lifespan must include concomitant adjustment to at least one other parameter to keep *R*_*0*_ constant. Numerical analyses are performed to assess how adjusting σ (the parasite death rate i.e. inverse of parasite lifespan) - while also adjusting in turn the parameters λ, *µ*_2_ or ψ in order to hold *R*_*0*_ constant - influences how many treatment rounds are required for elimination in the two compliance settings. Additionally we adjust *R*_*0*_ itself, while holding all parameters other than σ constant. This analysis is performed separately for a short- (*Ascaris lumbricoides*) or longer-lived (*Schistosoma mansoni*) parasites.

## Results

**Fig. 1:**
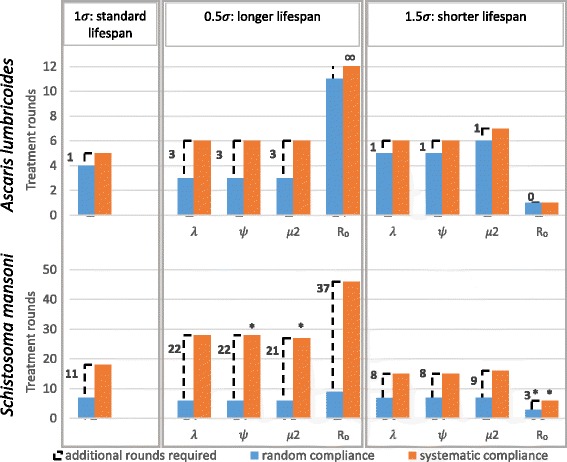
The minimum number of annual treatment rounds required for interruption of transmission. The number of annual MDA treatments required before the transmission breakpoint is crossed, in random compliance and systematic non-compliance settings. Treatment set at 75% coverage in all age groups. The parasite death rate σ has been decreased or increased by 50%, corresponding to a lengthened or shortened parasite lifespan, respectively. In turn each of the other key terms in the transmission intensity (*R*_*0*_) equation have been adjusted accordingly in order to assess how each influences the impact of the two different compliance patterns. **∞** elimination is impossible regardless of the number of annual treatment rounds. *after crossing the breakpoint infection remains for a long period (several decades) before eventual elimination

A systematic non-compliance setting, in which a portion of the population is never treated, increases the number of treatment rounds required before parasite transmission is interrupted. Our predictions here (Fig. 1) and elsewhere [8] are consistent with this conclusion. The number of additional treatment rounds required in simulation is a measure of the impact of systematic non-compliance under varying conditions.

We find a strong and consistent interaction between parasite lifespan and compliance setting, while controlling *R*_*0*_ by adjusting other parameters or allowing *R*_*0*_ itself to vary. This applies to both parasites studied. In all simulations a longer estimated parasite lifespan is associated with a much greater number of additional rounds required to overcome the effects of systematic non-compliance. A shorter lifespan is associated with fewer additional treatment rounds in the schistosome, and proportionally fewer in *Ascaris* (with respect to a slightly higher number of treatment rounds required for elimination in a random setting). *R*_*0*_ is the key factor in the model’s transmission intensity, and inspection of Eq. 1 indicates that adjustments to λ, ψ and *µ*_2_ should have comparable effects. This is the case; the slight discrepancy in outcome when adjusting *µ*_2_ is due to this parameter’s additional appearance elsewhere in the model [11].

Consistent with the predictions of a more complex stochastic individual-based implementation of this model we have already reported [8], we see a difference in behaviour between the two types of parasitic helminth infections. The impact of systematic non-compliance is much larger for the longer lived *Schistosoma mansoni* infection. In the presence of 25% systematic non-compliance, a higher estimate of this parasite’s lifespan would imply decades of annual treatment before the transmission breakpoint is reached (this study). In contrast, the difference between random and systematic non-compliance settings is generally rather lower for *Ascaris*, the shorter lived parasite. Though always a hindrance to elimination, the impact of systematic non-compliance is highly dependent on parasite species.

This strongly supports the conclusion that the difference in the impact of non-compliance between the two parasitic infections is due to the substantially longer schistosome parasite lifespan in the human host.

## Discussion

In general terms, compliance with treatment in patients suffering from chronic diseases averages 50% in developed countries, and is likely considerably lower in some developing countries with very poor healthcare systems and fewer resources [14]. A recent review of treatment uptake among the neglected tropical diseases covering STH, lymphatic filariasis, schistosomiasis, onchocerciasis, and trachoma found widely varying definitions of “compliance” and treatment coverage, and reported rates of compliance ranging from 19.5% to 99% [7].

Shuford et al. [7] note the much greater availability of individual compliance data with respect to MDA treatment for lymphatic filariasis and onchocerciasis, with a more limited number of studies of compliance with STH and schistosome treatment. Both Shuford et al. [7] and a recent review of community engagement in anti-malarial MDA [15] report concerns regarding inconsistent and unclear definitions of coverage and adherence. Estimates of systematic non-compliance require longitudinal surveys to ascertain treatment compliance at the individual level over successive rounds. In general, cost is a major disincentive within monitoring and evaluation programmes connected to MDA, and no such studies have yet been published with respect to STH or schistosomiasis. However, the need for these data has motivated collection of relevant individual-level longitudinal compliance information in the STH-focused DeWorm3 study currently being conducted [7].

Helminth infections that go untreated during an MDA programme may effectively create an inaccessible reservoir of infection in the human population that releases infective stages to sustain parasite transmission in the entire population. In the longer-lived helminth parasites such as schistosome and the filarial worms [12, 16] such an effect would be more pronounced given the longer parasite lifespan in the untreated people creating a sustained output of infective stages.

Predictions of an individual stochastic model indicate a potentially critical role for lifespan in interrupting parasite transmission when in combination with systematic non-compliance [8]. A variable impact of systematic non-compliance has also been identified in a stochastic model of lymphatic filariasis, in which impact is correlated with other factors including exposure to infective mosquito bites and use of insecticide-treated bednets [17]. In this paper we have extended these analyses to assess in detail how altering lifespan (and other parameters that contribute to transmission intensity) influences the effect of persistent non-compliance.

The average helminth parasite lifespan in the human host is difficult to measure accurately and published data compilations depend on fragmentary reports of egg excretion or microfilaremia post departure from endemic regions in the absence of reinfection [12]. Furthermore, an average lifespan in the human host does not provide information regarding a parasites’ individual developmental details such as time to maturation and hence egg production post-entry to the definitive host, or variation in egg production as worms age in the human host. With respect to modelling population-level transmission between hosts (who will generally be infected with a number of parasites), these details are crudely described in our models in the absence of more precise population biological data. Nevertheless, uncertainty over the length of these parasites’ lifespans in the human host adds to the need to take a close look at the sensitivity of model outcomes with respect to parameter assignments for this attribute.

We have shown that increases in parasite lifespan in the human host in a model of helminth transmission, do exacerbate the effect of systematic non-compliance with treatment in the population. Decreases to lifespan have the opposite effect, facilitating interruption of transmission. These effects persist despite accounting for concomitant changes elsewhere in the models’ parameters. Bearing in mind the difficulty of accurately estimating parasite lifespan in the human host, if these were somewhat longer than our default “standard” estimate it would be likely that interruption of *S. mansoni* transmission - when in the presence of even a modest level of systematic non-compliance - is substantially more difficult in a practicable time-frame than existing models would otherwise suggest.

Although a simplified, purely systematic compliance setting has been employed here in focussing on the transmission dynamics, more complex treatments of individual compliance somewhere between the two limits we examined are possible which give finer control over the precise degree of non-compliance over time [18, 19]. When data becomes available from studies such as DeWorm3 and TUMIKIA [4], measured compliance probability distributions derived from multiple rounds of MDA can be examined with respect to a variety of confounding effects such as age and gender.

## Conclusions

The sensitivity of the interaction between parasite lifespan and compliance with treatment requires that future modelling studies of anthelmintic MDA treatment impact take into account uncertainties around estimates of lifespan. Quantitative analyses here confirm a hypothesised strong interaction between helminth parasite lifespan in the human host and the impact of systematic non-compliance on parasite transmission. This suggests that the effectiveness of mass drug administration programmes targeting the longer-lived helminths (such as the filarial worms and the schistosome parasites) may be particularly improved through efforts to reduce systematic non-compliance. Further data, particularly longitudinal studies measuring individual compliance over multiple rounds of MDA stratified by age and gender, are critical as efforts are made towards elimination of STH and schistosomiasis. With this, and standardisation of terminology in reporting of MDA coverage and compliance, we can improve our understanding of the impact of current MDA based control programmes for the helminth NTDs and ultimately improve the design, monitoring and evaluation of public health measures.
